# Metabolic phenotypes and the gut microbiota in response to dietary resistant starch type 2 in normal-weight subjects: a randomized crossover trial

**DOI:** 10.1038/s41598-018-38216-9

**Published:** 2019-03-20

**Authors:** Lei Zhang, Yang Ouyang, Huating Li, Li Shen, Yueqiong Ni, Qichen Fang, Guangyu Wu, Lingling Qian, Yunfeng Xiao, Jing Zhang, Peiyuan Yin, Gianni Panagiotou, Guowang Xu, Jianping Ye, Weiping Jia

**Affiliations:** 1 Department of Endocrinology and Metabolism, Shanghai Jiao Tong University Affiliated Sixth People’s Hospital; Shanghai Key Laboratory of Diabetes Mellitus, Shanghai Clinical Center of Diabetes, Shanghai, 200233 China; 2 Department of Medicine, Shanghai Jiao Tong University School of Medicine, Shanghai, 200025 China; 3 CAS Key Laboratory of Separation Sciences for Analytical Chemistry, Dalian Institute of Chemical Physics, Chinese Academy of Sciences, Dalian, 116023 China; 4 University of Chinese Academy of Sciences, Beijing, 100049 China; 5 Department of Clinical Nutrition, Shanghai Jiao Tong University Affiliated Sixth People’s Hospital, Shanghai, 200233 China; 6 Leibniz Institute for Natural Product Research and Infection Biology, Hans Knöll Institute (HKI), Department of Systems Biology and Bioinformatics, Beutenbergstraße 11a, 07745 Jena, Germany; 7 Systems Biology & Bioinformatics Group, School of Biological Sciences and Department of Microbiology, The University of Hong Kong, Hong Kong, S.A.R. China; 8 Department of Radiology, Shanghai Jiao Tong University Affiliated Sixth People’s Hospital, Shanghai, 200233 China; 9 Antioxidant and Gene Regulation Laboratory, Pennington Biomedical Research Center, Louisiana State University System, Baton Rouge, Louisiana USA

## Abstract

Resistant starch (RS) has been reported to reduce body fat in obese mice. However, this effect has not been demonstrated in humans. In this study, we tested the effects of RS in 19 volunteers with normal body weights. A randomized, double-blinded and crossover design clinical trial was conducted. The study subjects were given either 40 g high amylose RS2 or energy-matched control starch with three identical diets per day throughout the study. The effect of RS was evaluated by monitoring body fat, glucose metabolism, gut hormones, gut microbiota, short-chain fatty acids (SCFAs) and metabolites. The visceral and subcutaneous fat areas were significantly reduced following RS intake. Acetate and early-phase insulin, C-peptide and glucagon-like peptide-1 (GLP-1) secretion were increased, and the low-density lipoprotein cholesterol (LDL-C) and blood urea nitrogen (BUN) levels were decreased after the RS intervention. Based on 16S rRNA sequencing, certain gut microbes were significantly decreased after RS supplementation, whereas the genus *Ruminococcaceae_UCG-005* showed an increase in abundance. Other potential signatures of the RS intervention included *Akkermansia*, *Ruminococcus_2*, *Victivallis*, and *Comamonas*. Moreover, the baseline abundance of the genera Streptococcus, *Ruminococcus_torques_group*, *Eubacterium_hallii_group*, and *Eubacterium_eligens_group* was significantly associated with the hormonal and metabolic effects of RS. These observations suggest that a daily intake of 40 g of RS is effective in modulating body fat, SCFAs, early-phase insulin and GLP-1 secretion and the gut microbiota in normal-weight subjects.

## Introduction

Dietary fibre is beneficial for the management of certain chronic diseases. Resistant starch (RS) is a prebiotic dietary fibre that is subject to fermentation by the gut microbiota in the intestine^1,2^. RS is reported to have beneficial effects on diabetes, obesity, inflammatory bowel disease, intestinal tumours, and cardiovascular diseases. The intake of RS per capita is 14.9 g/d in China^3^, 3 g/d per capita in Europe^4^, 3–8 g/d in the United States^5^, and 3–10 g/d^5^ worldwide on average, which are too low to have beneficial effects^6^.

RS has been reported to reduce body fat in obese rodents^7–12^. However, its effects have not been carefully tested in human subjects, especially in those with normal body weights. The effects of RS on the gut microbiota were reported in obese patients with dysbiosis and in healthy human subjects^13,14^, although its effects on body fat were not reported in those studies. Studies have shown that RS improves insulin sensitivity and glucose metabolism in overweight and obese subjects or patients with metabolic syndrome or type 2 diabetes, although no effects have been observed on body fat^15–19^. The current literatures suggest a discrepancy in the effects of RS on body fat between rodents and humans. Although no explanation is available for this discrepancy, differences in dietary components have been considered. Rodent studies have been conducted with controlled diets^7–12^ but not human studies^15–19^. The fatty acids in high-fat diets are known to inhibit RS fermentation in the large intestine^20,21^. Tea polyphenols inhibit fermentation by crosslinking amylose to generate large molecules beyond fermentable size^22^. Therefore, strict control of dietary components is required for clinical studies of RS.

In this study, we tested the effects of 40 g/d of RS on 19 normal-weight subjects on a controlled diet in a double-blinded and crossover clinical trial. The effects of RS on body fat, glucose, fatty acid metabolism, and the gut microbiota were examined. Our results suggest that RS reduces visceral and subcutaneous fat in normal-weight subjects and induces beneficial changes in gut hormones and the microbiota.

## Results

### Safety of RS supplementation

To exclude the interference of dietary components in this study, all subjects received a controlled diet with a standardized nutrient composition (Supplementary Table S1). No gastrointestinal adverse events, such as nausea, vomiting, bloating, or constipation were reported. Additionally, no serious adverse events that led to hospitalization or an inability to work were reported during the study period. To further assess the safety of RS supplementation, blood samples were collected for routine clinical chemistry and metabolomics analyses before and after RS and control starch (CS) administration. After 4 weeks of RS administration at 40 g/d, liver function indices, including the aspartate aminotransferase (AST), alanine aminotransferase (ALT), and γ-glutamyl transferase (GGT) levels, and renal function indices, such as the urea nitrogen and creatinine levels, were found to be within the normal ranges (Table 1). Amino acid and bile acid metabolism were also maintained within a normal and stable range (Supplementary Table S2), which confirmed the safety of RS supplementation.

**Table 1:** Anthropometric and biochemical assessments before and after 4 weeks’ RS or CS supplementation in normal-weight subjects.

Variable	CS		RS		*p* value
	0 week	4 weeks	0 week	4 weeks	
Weight (kg)	58.73 ± 2.44	58.37 ± 2.39	58.92 ± 2.39	58.78 ± 2.33	0.490
BMI (kg/m²)	21.08 ± 0.41	20.95 ± 0.42	21.14 ± 0.39	21.11 ± 0.37	0.423
FM (kg)	12.23 ± 0.90	12.04 ± 0.91	12.34 ± 0.88	12.33 ± 0.83	0.413
FFM (kg)	44.84 ± 3.33	46.39 ± 2.5	46.53 ± 2.41	46.46 ± 2.44	0.948
TBW (kg)	31.95 ± 1.67	32.39 ± 1.68	31.87 ± 1.58	31.93 ± 1.64	0.502
Fat percentage (%)	21.29 ± 1.72	21.12 ± 1.75	21.41 ± 1.67	21.51 ± 1.65	0.826
WC (cm)	72.08 ± 1.32	71.84 ± 1.28	72.76 ± 1.47	72.09 ± 1.37	0.943
HC (cm)	93.00 ± 1.06	92.89 ± 0.97	93.57 ± 1.09	92.98 ± 1.18	0.476
WHR	0.78 ± 0.01	0.77 ± 0.01	0.78 ± 0.01	0.78 ± 0.01	0.560
VFA (cm²)	26.04 ± 2.52	27.05 ± 2.67*	26.43 ± 2.63	21.70 ± 1.78***^###^	<0.001
SFA (cm²)	134.18 ± 10.19	134.65 ± 10.18	135.81 ± 10.57	127.33 ± 10.66**^#^	0.031
ALT (U/l)	12.58 ± 1.66	11.63 ± 1.30	11.79 ± 1.45	12.58 ± 1.79	0.208
AST (U/l)	17.74 ± 1.67	16.21 ± 0.71	16.21 ± 0.85	15.95 ± 1.00	0.379
GGT (U/l)	13.32 ± 1.21	13.74 ± 1.51	14.26 ± 1.37	13.68 ± 1.07	0.395
TC (mmol/l)	4.41 ± 0.19	4.48 ± 0.16	4.38 ± 0.17	4.29 ± 0.17^#^	0.140
TG (mmol/l)	0.76 ± 0.08	0.80 ± 0.07	0.76 ± 0.08	0.78 ± 0.06	0.873
HDL-C (mmol/l)	1.40 ± 0.07	1.38 ± 0.06	1.36 ± 0.07	1.31 ± 0.05	0.515
LDL-C (mmol/l)	2.59 ± 0.12	2.73 ± 0.12*	2.62 ± 0.11	2.57 ± 0.14^#^	0.006
BUN (mmol/l)	4.11 ± 0.25	4.10 ± 0.28	4.24 ± 0.25	3.66 ± 0.19*^#^	0.010
Cr (μmol/l)	66.05 ± 2.87	68.32 ± 2.54	67.42 ± 2.59	68.84 ± 2.94	0.633
UA (μmol/l)	292.32 ± 12.58	286.95 ± 14.63	305.58 ± 17.22	284.63 ± 13.48*	0.183

Data are presented as mean ± SEM. FM: fat mass; FFM: fat-free mass; TBW: total body water; WC: waist circumference, HC: hip circumference, WHR: Waist hip ratio, VFA: Visceral fat area, SFA: Subcutaneous fat area, TG: triglyceride level; HDL-C: high-density lipoprotein cholesterol. *p* value was statistical significance between CS effect and RS effect; Significance was determined by generalized estimating equation (GEE) model; *p < 0.05, **p < 0.01, starch 0 week vs. starch 4 weeks, ^#^*p* < 0.05, ^###^*p* < 0.001, RS 4 weeks vs. CS 4 weeks.

### RS supplementation reduced visceral subcutaneous and intra-abdominal fat

**Figure 1:**
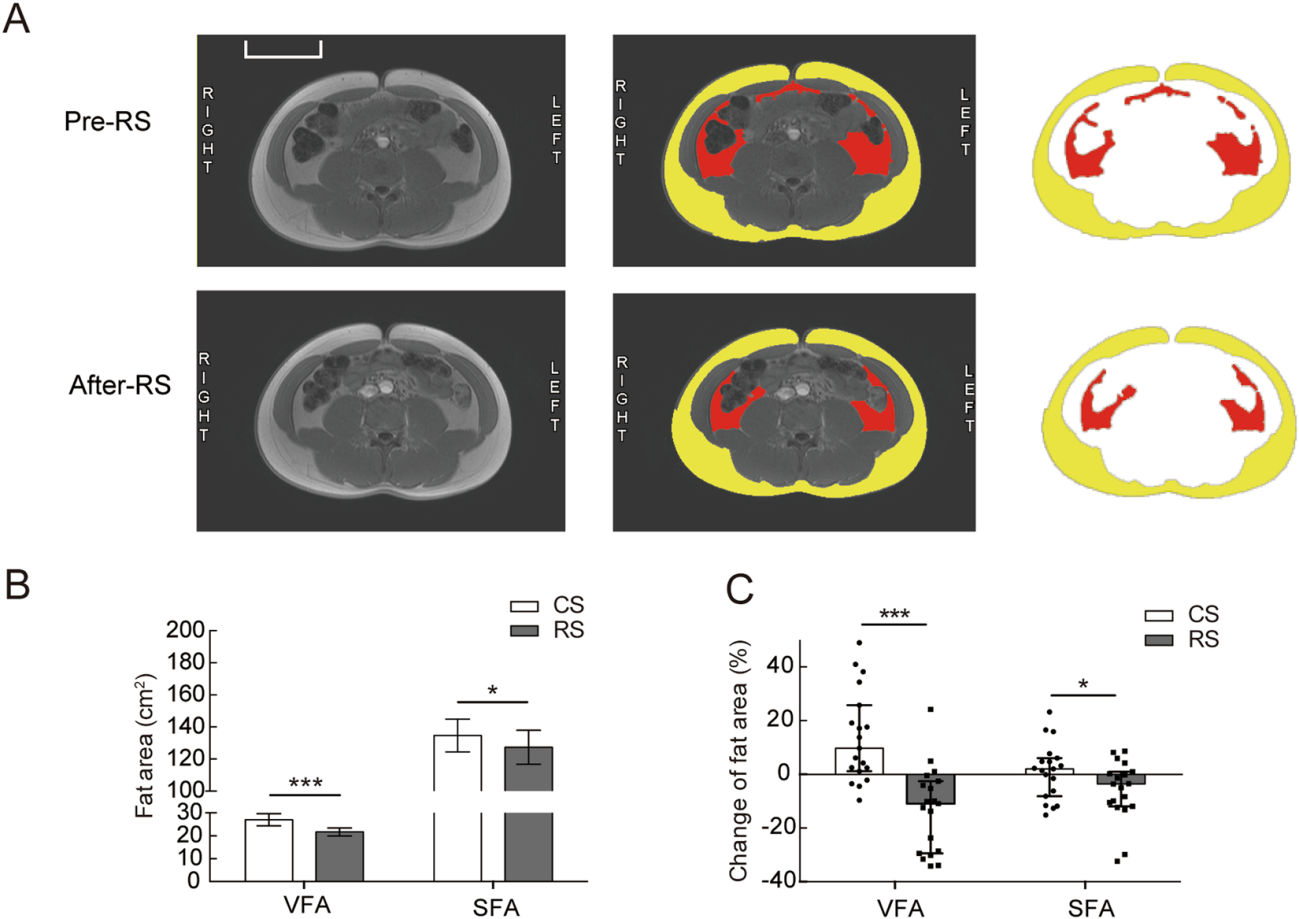
Body composition before and after 4 weeks of RS or CS supplementation in normal-weight subjects. Nineteen normal-weight subjects were followed up for 4 weeks of dietary supplementation with RS or CS in a crossover study. (**A**) Representative abdominal MRI of the subjects before and after 4 weeks of RS consumption. Raw (left panel) and marked (middle and right panel) MRI at the navel level; yellow delineates the SFA and red delineates the VFA. (**B**) The SFA and VFA in the participants after a standardized meal following 4 weeks of consumption of RS or CS (n = 19). Data are presented as the mean ± SEM. (**C**) Changes in the fat area (%) (SFA and VFA) evaluated by MRI in subjects after 4 weeks of CS or RS intake (n = 19). Data are presented as the median (interquartile range), Significance was determined using the GEE model. Scale bar = 10 cm. *p < 0.05, ***p < 0.001.

RS intake significantly reduced abdominal adiposity (visceral fat area [VFA] and subcutaneous fat area [SFA]) (Fig. 1A). The VFA showed a significant reduction after RS intake compared to that after CS consumption (27.05 ± 2.67 cm² vs. 21.70 ± 1.78 cm², p < 0.001) (Fig. 1B), whereas no difference was observed in the baseline levels between the two starch intake groups (Table 1). A reduction was also observed in the SFA (Fig. 1B). The SFA and VFA were decreased by −10.88% (−28.96%, −3.30%) and −3.53% (−11.21%, 0.6%), respectively (Fig. 1C). However, no significant change in body weight was observed after RS supplementation (Table 1).

We found significant decreases in the low-density lipoprotein cholesterol (LDL-C) (2.73 ± 0.12 vs. 2.57 ± 0.14 mmol/l, p < 0.05) and blood urea nitrogen (BUN) (4.10 ± 0.28 mmol/l vs. 3.66 ± 0.19 mmol/l, p < 0.05) levels in the RS group compared with those of the CS group. Uric acid (UA) was also significantly reduced after RS intake (p < 0.05). No significant changes were found in the ALT, AST, and GGT levels (Table 1). The anthropometric parameters, including the body mass index (BMI), whole body fat mass and percentage, waist circumference, hip circumference, and waist-to-hip ratio, were not significantly changed after RS or CS supplementation. Furthermore, we found that metabolites related to β-oxidation of long-chain fatty acids were reduced in the urine following RS treatment, including carnitine C2:0, carnitine C8:0, and carnitine C8:1 (Supplementary Fig. S1).

### RS supplementation promotes secretion of early-phase insulin, gut hormones and SCFAs

**Figure 2:**
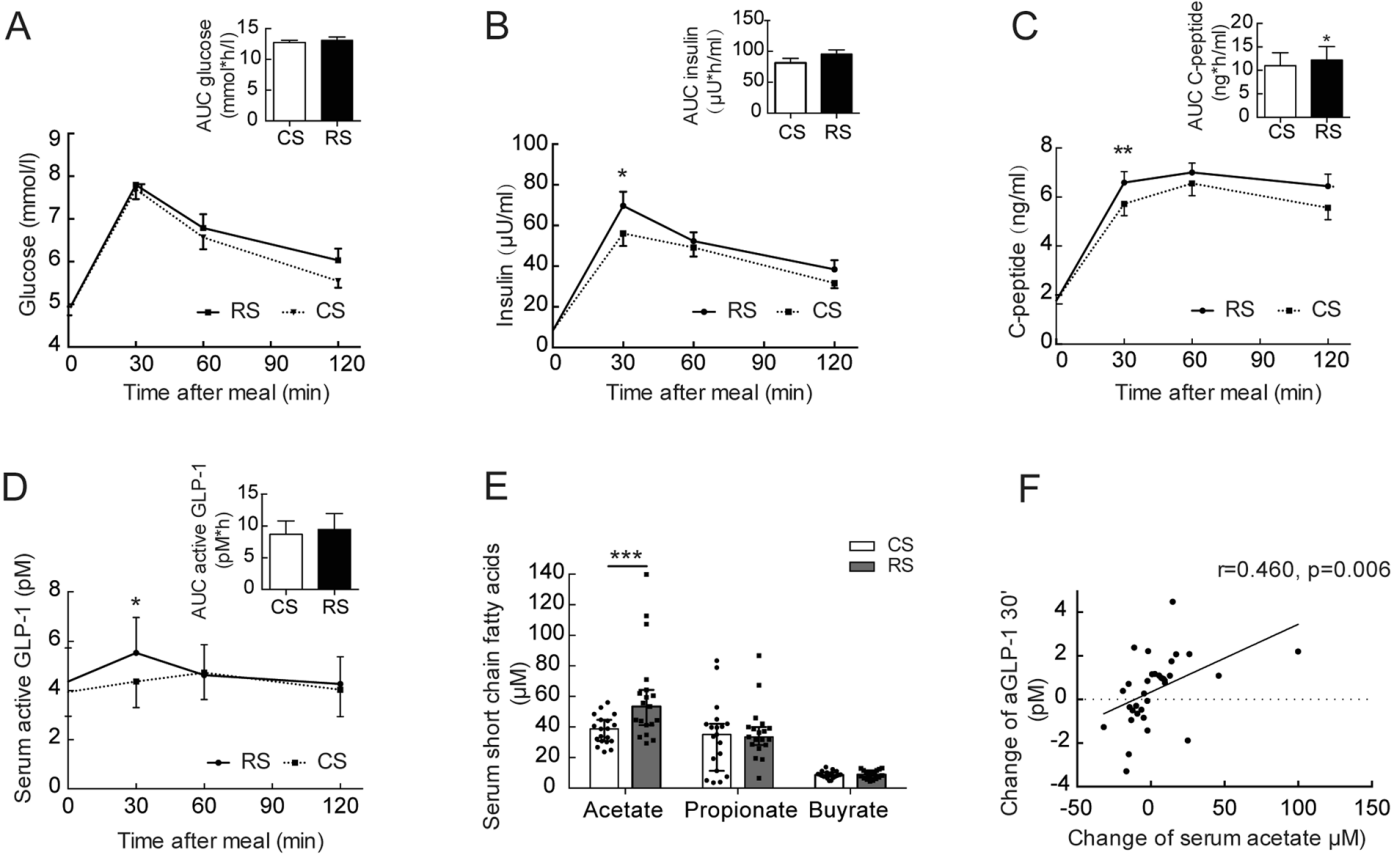
Gut hormones and SCFAs after 4 weeks of RS or CS supplementation in normal-weight subjects. (**A**) Blood glucose levels and the AUC, (**B**) blood insulin levels and the AUC, (**C**) blood C-peptide levels and the AUC, and (**D**) blood aGLP-1 levels and the AUC after a standardized meal in participants after 4 weeks of RS or CS supplementation. (**E**) Serum SCFA levels in the participants after 4 weeks of RS or CS supplementation. Data are presented as the mean ± SEM. The aGLP-1 and SCFA data were log transformed before the analysis. Significance was determined with the generalized estimating equation model. *p < 0.05, ***p < 0.001. (**F**) Relationship between the changes in acetate and the changes in aGLP-1 at 30 min after starch supplementation.

The effect of RS intake on glucose metabolism was examined in two tests: the standard meal tolerance test and the hyperinsulinemic-euglycemic clamp. In the meal tolerance test, the fasting and postprandial glucose levels were not altered by RS intake (Fig. 2A). However, at 30 min after the meal, a significant increase in the insulin (56.98 ± 7.57 μU/ml vs. 69.70 ± 6.90 μU/ml, p < 0.05) and C-peptide (5.72 ± 0.48 ng/ml vs. 6.59 ± 0.45 ng/ml, p < 0.01) levels was seen after RS intake compared to that after CS consumption, and the area under the curve (AUC) for C-peptide was significantly higher after RS intake than after CS consumption (Fig. 2B,C). In contrast, no difference was observed in the baseline levels between the two starch intake groups (Supplementary Table S3). In the hyperinsulinemic-euglycemic clamp test, no significant impact of RS was observed on the glucose infusion rate (Supplementary Table S3). Therefore, the data suggest that early insulin secretion is enhanced by RS supplementation.

To understand the basis of this early insulin secretion, we examined the serum levels of gut hormones [glucagon-like peptide-1 (GLP-1) and peptide YY (PYY)] and short-chain fatty acids (SCFAs). A significant increase was observed in active GLP-1 (aGLP-1) at 30 min (*p* < 0.05) in the meal tolerance test after RS intake compared to that after CS consumption (Fig. 2D). The changes in GLP-1 disappeared at 60 and 120 min following the meal tolerance test (Fig. 2D). RS consumption did not significantly affect the PYY level (Supplementary Fig. S2). Furthermore, the serum short-chain fatty acid acetate was significantly increased after RS intake compared to that after CS consumption [53.34 (41.58,63.18) μM vs. 38.79 (30.81,44.38) μM, *p* < 0.001], whereas the levels of the other SCFAs (propionate, butyrate, isobutyrate, valerate, isovalerate and hexanoate) did not change after RS intake (Fig. 2E, Supplementary Table S2). Furthermore, the change in acetate after starch consumption was positively correlated with the postprandial change in aGLP-1 at 30 min (r = 0.460, *p* = 0.006, Fig. 2F).

### RS intake changes the gut microbiota composition in adults with normal weights

**Figure 3:**
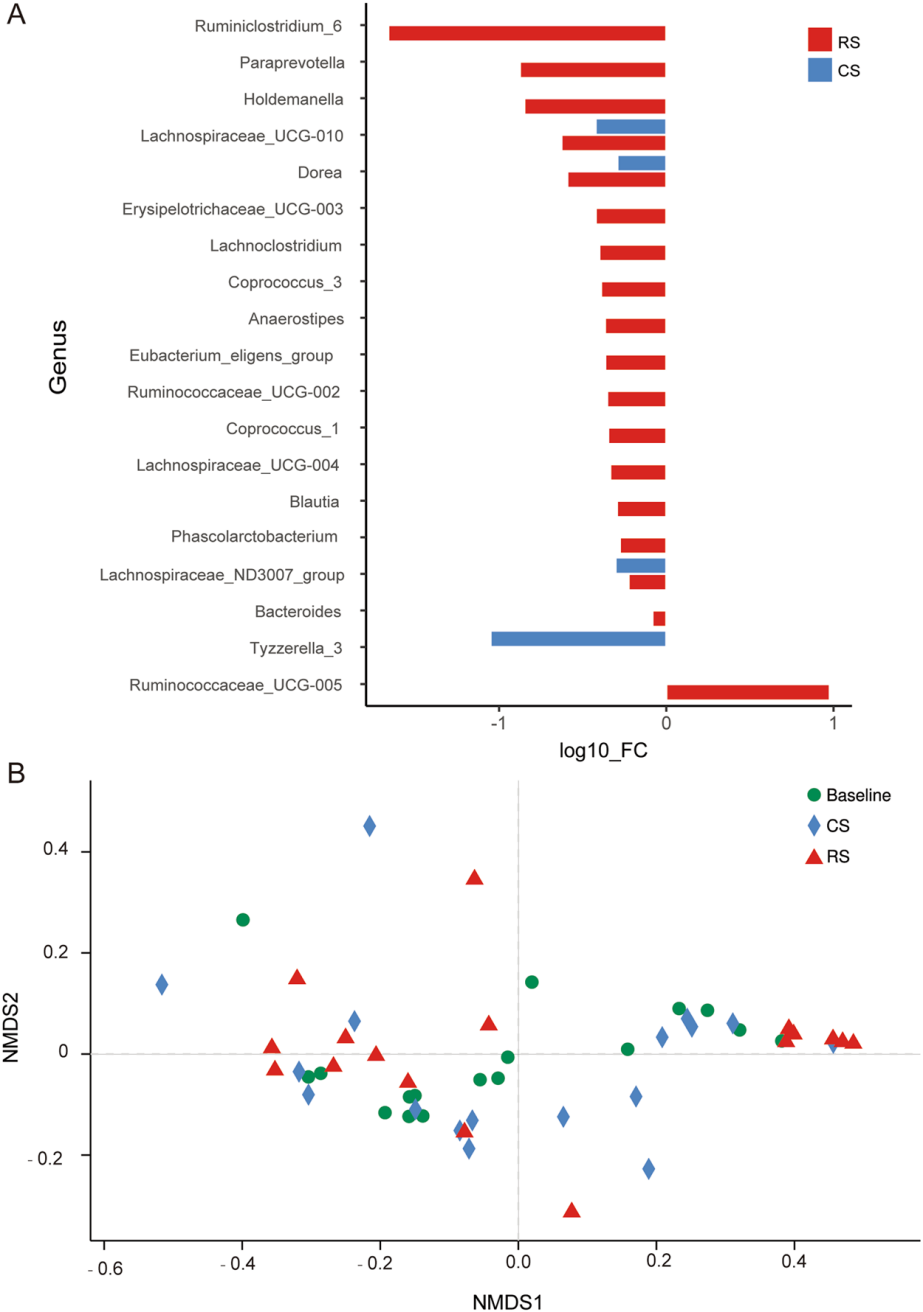
The changes in the gut microbial composition after RS supplementation at the genus level. (**A**) Differences in bacterial abundance at the genus level were analysed before and after 4 weeks of RS or CS intake in normal-weight individuals. Only genera whose abundances were significantly changed are shown (*p* < 0.05, Wilcoxon signed-rank test, n = 17). The mean abundances of all samples at baseline or after the intervention were used to calculate FC (fold changes) (and then log transformed). If no significance was detected, the fold change was set to 1. (**B**) Non-metric multidimensional scaling (NMDS) ordination plot based on genus-level microbial beta diversity (UniFrac distances).

The gut microbiota composition was examined in 17 participants using the 16S rRNA sequencing protocol to understand the mechanism underlying the GLP-1 and PPY alterations in the RS group. A total of 537,298 original sequences were obtained from 51 samples. An operational taxonomic unit (OTU)-based community-level analysis revealed no significant differences in the alpha diversity indices (Shannon, Chao1 and PD_whole_tree) between the values at baseline and after 4 weeks of RS and CS intake (Supplementary Fig. S3). At the phylum level, the composition did not differ among the three groups. *Bacteroidetes* was the most dominant phylum in all groups, followed by *Firmicutes* and *Proteobacteria* (Supplementary Fig. S4). Then, we investigated the effect of RS on the differences in the gut microbiota composition at the genus level. We found that the levels of fifteen bacterial genera were significantly decreased specifically after RS intake relative to the baseline levels (Fig. 3), which included *Anaerostipes*, *Bacteroides*, *Blautia*, *Holdemanella*, *Coprococcus_1*, *Coprococcus_3*, *Lachnoclostridium*, *Lachnospiraceae_UCG-004*, *Erysipelotrichaceae_UCG-003*, *Paraprevotella*, *Phascolarctobacterium*, *Ruminiclostridium_6*, *Ruminococcaceae_UCG-002*, and *Eubacterium_eligens_group*. In addition, RS intake significantly increased the abundance of genus *Ruminococcaceae_UCG-005*. The bacterial genera *Lachnospiraceae_UCG-010, Doera* and *Lachnospiraceae_ND3007_group* were decreased in both the RS and CS groups (Fig. 3A). Comparison of the overall communities using non-metric multidimensional scaling (NMDS) with weighted UniFrac distances showed that the samples did not cluster together according to their groups (Fig. 3B). Furthermore, an increasing trend of abundance of *Akkermania* and *Victivallis* was observed after RS intake compared to that at baseline (*p* = 0.059 and *p* = 0.059, respectively). RS also led to significant abundance changes for a range of bacterial OTUs (Supplementary Figs S5, S6). In addition, metabolites of the gut microbiota, such as N-acetyl-DL-tryptophan and indole lactic acid, were significantly increased in the urine after RS intake (Supplementary Fig. S2).

**Figure 4:**
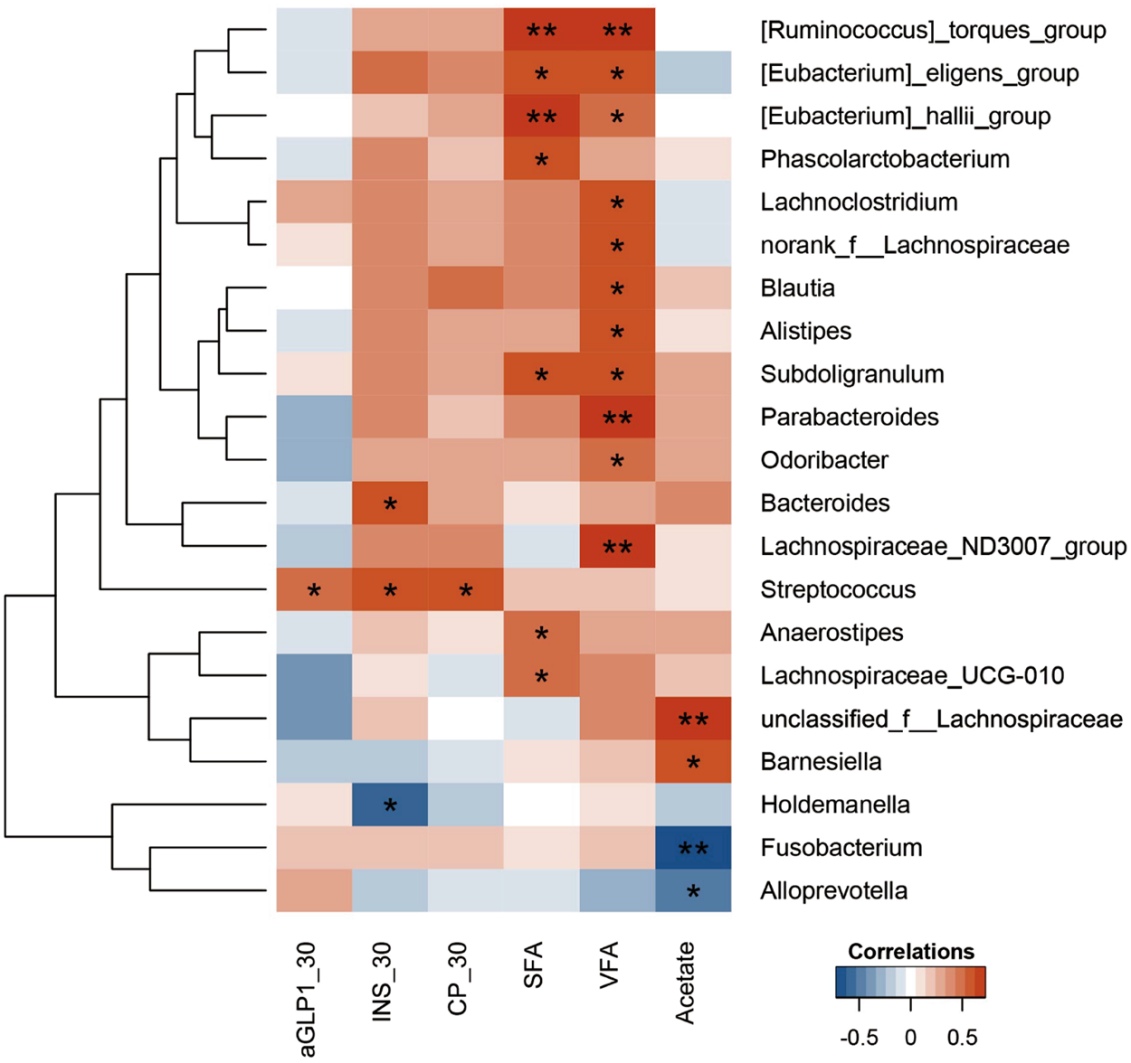
The correlation heatmap of the baseline gut microbiota with altered host phenotypes and SCFAs. Spearman’s correlations between the abundances of the top 50 most abundant bacterial genera at baseline and the changes in the phenotypes or SCFAs after RS intake were calculated. Blue: negative correlations; red: position correlations. *p < 0.05, **p < 0.01. aGLP1_30: postprandial aGLP-1 at 30 min; INS_30: postprandial insulin at 30 min; CP_30: postprandial C-peptide at 30 min.

To study the clinical impact of the gut microbiota, we analysed the correlations of the microbiota at baseline and the changes seen in the hormone levels, such as the changes in postprandial aGLP-1, insulin and C-peptide at 30 min, acetate, and the SFA and VFA after RS supplementation. Hormones at 30 min postprandial were elevated by RS and were all correlated with a high baseline abundance of *Streptococcus*. Additionally, the change in acetate after RS intake was positively correlated with *Barmesiella* and *unclassified_f_Lanchnospiraceae* and negatively correlated with *Fusobacterium* and *Alloprevotella*. In addition, the decreases in the SFA and VFA after RS supplementation were both correlated with a low baseline abundance of *Ruminococcus_torques_group*, *Eubacterium_hallii_group*, and *Eubacterium_eligens_group* (Fig. 4).

## Discussion

In this placebo-controlled, double-blinded, and crossover trial, RS was found to reduce abdominal adiposity in normal-weight subjects. Intra-abdominal visceral and abdominal subcutaneous fat were significantly reduced by taking RS at 40 g/d in the 4-week study. RS has been reported to have no effect on body fat in patients with metabolic syndrome or type 2 diabetes mellitus^15–19^. In those studies, the whole body weight and fat mass were used as indicators of body fat, which might have led to a misinterpretation of data for at least two reasons. First, the fat reduction from RS intake might have been negated by an increase in the intestinal weight, which was not distinguishable from the total body weight. RS is known to increase the tissue weight of the large intestine through a hypertrophic effect on the intestinal wall^8,23^. Second, diet components were not controlled in these studies, and the dietary impact on the results was not considered. Moreover, interference with the effects of RS by dietary components, such as fatty acids^21^, tea polyphenols^22^ and unknown components, may have contributed to the negative results. In the current study, these two issues were addressed using fat-specific analysis and a controlled diet for all subjects. Body fat was measured using a more accurate MRI method that detected changes in the abdominal fat area regardless of alterations in the whole body weight. The fat reduction was observable even in the absence of a reduction in the whole body weight in our study. In addition, we controlled dietary components by providing an identical diet to all subjects. Nonetheless, our nutrient intake data are based on self-reported dietary records, and subjective bias should be considered.

Our data suggest that GLP-1 may play a role in mediating the effects of RS. We observed that the serum GLP-1 level was elevated by RS intake in normal-weight subjects. The elevation in the gut hormone levels provides a mechanism for the effects of RS on abdominal adiposity and early-phase insulin secretion. GLP-1 can increase energy metabolism through its effects on the brain and by promoting insulin secretion through direct stimulation of β cells^24,25^. Our results are consistent with those observed in rodents for induction of GLP-1 and PYY by RS^11,26^. The mechanism may be related to the chronic effects of RS on L-cells, which in turn are related to stimulation of GLP-1 secretion by butyrate through G protein-coupled receptor 43 (GPR43)^27^. RS can also induce L-cell numbers through these chronic effects. The fermentation products of digestion are required for maintenance of mucosal integrity in the colon^8,23^. Damage to mucosal integrity may lead to a reduction in L-cell numbers. The chronic effects were not examined in these acute studies^28–30^, which might have contributed to the reduction in GLP-1.

RS was previously found to increase faecal SCFAs, including acetate, propionate and butyrate, in rats^8^. A significant increase in the serum SCFA acetate level was also found following RS intervention in our study. Moreover, the change in acetate after starch consumption was positively correlated with the postprandial change in aGLP-1 at 30 min. SCFAs in the microbiota act as signalling molecules and nutrients in the colon^31^. Additionally, SCFAs can be transported from the intestinal lumen into the peripheral blood^32^, where they affect lipid, glucose and cholesterol metabolism and are taken up by organs^33–35^. Therefore, our data suggest that RS may induce hormone secretion through stimulation of fermentation and SCFAs.

The gut microbiota was not changed at the phylum level by RS, which was consistent with the findings of a previous report in healthy human subjects^13^. However, RS intake significantly increased the abundance of *Ruminococcaceae_UCG-005*, decreased the abundance of fifteen genera, including *Bacteroides, Anaerostipes, Blautia, Holdemanella, Coprococcus_1, Coprococcus_3, Lachnoclostridium, Lachnospiraceae_UCG-004, Erysipelotrichaceae_UCG-003, Paraprevotella, Phascolarctobacterium, Ruminiclostridium_6, Ruminococcaceae_UCG-002*, and *Eubacterium_eligens_group*, and increased the trend in the abundance of *Akkermania* and *Victivallis*. RS2 intake has been reported to increase the levels of the species *Ruminococcus bromii* and *Eubacterium rectale*^13^. However, we did not observe the same results, possibly because of the limitation in the sensitivity of the 16S sequencing method used in our study.

*Bacteroides* is a predominant genus within the human intestine and was decreased following RS intake in our study. The effect is similar to that of blackcurrant, dietary α-cyclodextrin and soluble dietary fibre^36–38^. A decrease in *Bacteroides* sp. was also observed in overweight or obese children after oligofructose-enriched inulin treatment^39^, and a similar change was reported in rats after walnut treatment^40^. *Anaerostipes* is a genus of anaerobic bacteria from the *Lachnospiraceae* family, which was also decreased by RS in this study. The decrease in the *Anaerostipes* levels was also observed in another human study on inulin-type fructan intake^41^. *Blautia*, which is another genus of the *Lachnospiraceae* family, was decreased by RS in this study, which was consistent with observations in healthy Caucasian subjects treated with a walnut-enriched diet^42^. The reductions in *Coprococcus*, and *Ruminococcus* (*Lachnospiraceae* family) in our study were consistent with those reported in subjects consuming dietary fibre^43^. *Ruminococcaceae_UCG-005*, which is a member of family *Ruminococcaceae* that has been reported to attenuate dietary obesity^44^, was significantly increased by RS in this study. *Akkermansia muciniphila*, which is a species of genus *Akkermansia*, was reported to improve metabolic disorders in dietary obese mice^45^, and an increasing trend of the *Akkermansia* genus was also found following RS intake in this study. Therefore, our data suggest that changes in the gut microbiota at the genus level may contribute to the beneficial effects of RS.

We found, for the first time, that the baseline level of genus *Streptococcus* might mediate the effects of RS on hormones and abdominal fat. Increases in the postprandial levels of active GLP-1, insulin, and C-peptide were associated with a high abundance of genus *Streptococcus* at baseline in the RS group. This observation was surprising, because an increase in *Streptococcus* was previously shown to be associated with obesity^46^. Most species in genus *Streptococcus* are non-pathogenic, although some are pathogenic. Furthermore, low levels of *Ruminococcus_torques_group*, *Eubacterium_hallii_group*, and *Eubacterium_eligens_group* at baseline were positively associated with reductions in the VFA and SFA in this study, suggesting that baseline levels of *Ruminococcus_torques_group* are beneficial for the control of body fat. However, a decrease in *Ruminococcus_torques_group* was reported in a study that evaluated the beneficial effects of the probiotic *Akkermansia muciniphila* in non obese diabetic mice^47^. Nonetheless, to obtain further details regarding changes in bacterial genus levels after RS intake, whole-metagenome sequencing, faecal microbiota transplant and a functional study of specific bacteria are warranted in future studies.

In conclusion, RS intake (4 weeks of 40 g/d) significantly reduced the intra-abdominal and subcutaneous fat areas, increased hormone secretion (early-phase insulin, C-peptide, and GLP-1), decreased the levels of several gut bacteria genera, including *Anaerostipes, Bacteroides, Blautia*, and *Holdemanella*, and increased that of genus *Ruminococcaceae_UCG-005* in normal-weight adults. A higher abundance of the genus *Streptococcus* promotes an increase in hormones 30 min postprandial by RS, and the lower abundances of *Ruminococcus_torques_group*, *Eubacteriu_hallii_group* and *Eubacterium_eligen_group* promote the effects of RS on abdominal adiposity. The intervention did not lead to any adverse events in the subjects, as indicated by the blood and urine metabolomics. This study provides a basis for the safety and efficacy of RS type 2 supplementation at 40 g/d for the control of abdominal adiposity and glucose metabolism.

## Methods

### Study subjects and design

Eligible subjects were Chinese individuals between the ages of 18 and 55 years with a BMI < 24 kg/m^2^ and a waist circumference <85 cm in men and <80 cm in women. The exclusion criteria were as follows: acute or chronic diseases; probiotic supplement or antibiotic use 3 weeks before the study; participation in other clinical trials 4 weeks before the study; medication use that might affect glucose and lipid metabolism; and participation in organized physical activities. The subjects were advised to maintain their current physical activity level throughout the study. Twenty-two subjects with normal weights were screened, and nineteen were finally enrolled between July 2013 and July 2015 from the city of Shanghai, China. The study was conducted in a randomized, double-blind, crossover, and placebo-controlled manner. After enrolment, the subjects were divided randomly into two groups and underwent a 1-week run-in period. A uniform diet was designed and provided by the Department of Nutrition of Shanghai Jiao Tong University Affiliated Sixth People’s Hospital to ensure that all subjects received almost the same food with equal overall macronutrients and caloric intake during the whole process, from the run-in period to the end of the trial. Our uniform diet was designed in line with the Chinese dietary guideline, with 55–60% of the total calories from carbohydrates, ~25% from fat, and 15–20% from protein^48^. Three consecutive 24-hour dietary records (2 week days and 1 weekend day) at baseline and at the end of each period were required for each subject. The diet information was collected and analysed using a nutrition treatment computing system (NCCW, Qingdao, China). The subjects consumed either HAM-RS2 (Ingredion Inc., Bridgewater, NJ, USA) at 255.4 kcal/day (2.8 kcal/g, 91.2 g, containing 40 g of RS) or matched control starch (Ingredion Inc.) at 255.6 kcal/day (3.55 kcal/g, 72 g, amylopectin, containing 0 g of RS) alternately for four weeks separated by a four-week washout period in the crossover study. The order of starch supplementation was blinded for both the investigator and the participants. An independent researcher performed permuted block randomization and assigned the subjects to the starch supplementations. Computer-generated random numbers were used to assign the subjects in pairs to first receive either RS or CS. The RS and CS were packaged in sealed bags that were identical in appearance, and the subjects and investigators were unaware of the contents of the study starch and the randomization scheme. During the trial, all subjects were provided an identical diet without a change in their exercise habits. Three subjects withdrew from the study before the first intervention, and 19 volunteers, including 10 women and 9 men, completed the study. The data and samples were collected from the subjects through clinical check-ups before and at the end of each intervention.

The study was performed in accordance with the Declaration of Helsinki and was approved by the Ethics Committee of Shanghai Jiao Tong University Affiliated Sixth People’s Hospital. Written informed consent was obtained from all subjects. The trial was registered at the World Health Organization International Clinical Trials Registry Platform with the identification number ChiCTR-TTRCC 13003333 on July 3, 2013.

### Anthropometric and biochemical assessments

During the clinical check-ups, the subjects came to the Shanghai Clinical Centre of Diabetes by car or bus in the morning after an overnight (>10 hr) fast. Anthropometric data and venous blood, urine and faecal samples were collected. The waist circumference (WC) was measured at the middle between the lower border of the rib cage and the top of the lateral border of the iliac crest at the end of an expiratory breath. The hip circumference (HC) was measured at the widest part over the greater trochanters by the same researcher. The waist-to-hip ratio (WHR) was calculated. The body composition was measured with the TBF-410 Tanita Body Composition Analyser (Tanita, Tokyo, Japan).

Serum samples were collected and stored at −80 °C prior to the analysis. Fasting plasma glucose (FPG) and postprandial glucose were measured using the standard glucose oxidase method. The blood chemistry parameters included serum AST, ALT, GGT, serum creatinine, blood urea nitrogen (BUN), uric acid (UA), total cholesterol (TC), triglycerides (TGs), high-density lipoprotein cholesterol (HDL-C), and low-density lipoprotein cholesterol (LDL-C), which were measured by enzymatic procedures using an autoanalyser (Hitachi 7600–020 automatic analyser, Tokyo, Japan) according to the manufacturer’s instructions. Fasting and postprandial serum insulin and C-peptide were measured with an electrochemiluminescence immunoassay on a Cobas e411 analyser (Roche Diagnostics GmbH, Mannheim, Germany). Serum active GLP-1 (aGLP-1) and total PYY were measured using quantitative ELISA kits (Millipore, Darmstadt, Germany).

### Meal tolerance test (MTT)

To assess glucose metabolism, venous blood samples were taken at serial time points from the subjects following an MTT with instant noodles (1566.6 KJ; 68.4 g carbohydrates and 10.4 g protein). The collection included fasting and postprandial states. Glucose and insulin were examined in the blood as described above.

### Hyperinsulinaemic-euglycaemic clamp

Insulin sensitivity was assessed in subjects with a hyperinsulinaemic-euglycaemic clamp as previously described^49^. The insulin level was maintained at 100 μU/ml by prime-continuous infusion of insulin. The blood glucose concentration was maintained constantly at the basal level by adjusting the glucose infusion rate using the negative feedback principle.

### Magnetic resonance imaging

The body fat content was assessed in visceral and subcutaneous adipose tissue areas using a 3.0 T clinical MRI scanner (Archiva, Philips Medical System, Amsterdam, The Netherlands). The standard array coils were used, and the MRI scans were performed by experienced radiologists who were blinded to the intervention groups and laboratory findings. The MRI scans were conducted at the abdominal level between the L4 and L5 vertebrae in the supine position. Segmentation of the images into the VFA and SFA was performed by two trained investigators using the SliceOmatic image analysis software (version 4.2; Tomovision Inc., Montreal, QC, Canada).

### Gastrointestinal symptoms

After each treatment in this study, the subjects were given printed sheets to record the incidence and magnitude of GI responses and the details of their bowel movements after consumption of the test starch. Responses to questions on nausea, borborygmi (audible bowel sounds), colic, bloating and flatulence were recorded. The subjects recorded the number of bowel movements and the consistency of their faeces (normal, hard or watery). Information regarding the faecal volume and weight was not gathered.

### Metabolomics profiling of human serum and urine samples

Quality control (QC) samples, which were prepared by mixing and blending equal volumes (10 µL) of each sample, were used to estimate a mean profile representing all analytes encountered during the analysis. First, 50 µl of the serum, urine and QC samples were precipitated with 200 µl of methanol in 96-well plates. After vortexing, the filter liquor was obtained via positive pressure and then freeze-dried. The samples were analysed by ultra-performance liquid chromatography (Waters, USA) coupled to a Triple TOF 5600 mass spectrometer (AB SCIEX, USA) system. Pooled QC samples for every ten samples were injected during sample detection to monitor system stability.

Under LC conditions, solvent A was 0.1% (v/v) formic acid/water and solvent B was 0.1% (v/v) formic acid/methanol. The flow rate was set to 0.4 ml/min. For serum sample analysis, each sample was re-dissolved with 50 μl of methanol/water (1:4) solvent. The chromatography column used was a Waters BEH C8 (1.7 μm, 2.1 × 50 mm). The column temperature was 60 °C. The LC gradient was set as follows: 5% B at 0 min and maintained to 0.5 min, 40% B at 2 min, 100% B at 8 min, 100% B at 10 min, 5% B at 10.1 min and maintained for 1.9 min. For urine sample analysis, each sample was re-dissolved with 120 μL of acetonitrile/water (5:95) solvent. The chromatography column was a Waters HSS T3 (1.8 μm, 2.1 × 50 mm). The column temperature was 50 °C. The LC gradient was set as follows: 5% B at 0 min and maintained to 0.5 min, 50% B at 6 min, 90% B at 7 min, 90% B at 8 min, 5% B at 8.1 min and maintained for 1.9 min.

The serum and urine analyses were conducted with the same MS conditions. The mass scanning range was set at 60–1000 m/z for dd-MS2 mode. The sheath gas and curtain gas were set at flow rates of 55 and 35 psi, respectively. The spray voltage was 5.5 kV and the capillary temperature was 550 °C in positive ion mode, whereas the spray voltage was −4.5 kV and the capillary temperature was 450 °C in negative ion mode.

### SCFAs in human serum samples

First, 100 µL of serum was mixed with 35 µL of concentrated sulfuric acid diluted with 50% water. Then, 165 µL of ether containing an internal standard was added, mixed thoroughly by vortexing for 1 min, centrifuged at 12,000 rpm for 20 min at 4 °C, and left standing at 4 °C for 30 min. The supernatant ether layer was removed over anhydrous sodium sulfate, which was added to remove traces of water. The resultant supernatant ether layer was transferred to a GC autosampler vial, and short-chain fatty acids were quantitatively determined by gas chromatography (Agilent, USA). The reagent preparation procedure and temperature gradient for the SCFA analysis were adapted from Zheng X. *et al*.^50^.

### Faecal sample collection and DNA extraction

Fresh faecal samples were collected using a commercial tube with DNA stabilizer (STRATEC Molecular GmbH, Berlin, Germany) before and after each intervention and stored at −80 °C prior to analysis. Stool DNA was extracted using the E.Z.N.A. Soil DNA kit (Omega Bio-tek, Norcross, GA, USA) according to the manufacturer’s instructions.

### 16S rRNA sequencing

The V3 region of the 16S ribosomal RNA (rRNA) gene from each DNA sample was amplified using the bacterial universal forward primer 5′-AGAGTTTGATCCTGGCTCAG-3′ and the reverse primer 5′-TTACCGCGGCTGCTGGCAC-3′. The PCR reaction was conducted under the following conditions: 95 °C for 2 min, 25× (95 °C for 30 s, 55 °C for 30 s, and 72 °C for 30 s) and 72 °C for 5 min. Roche GS FLX Titanium emPCR kits were used for the PCR amplification, Sequencing was carried out using an Roche Genome Sequencer with the Roche GS FLX + Sequencing Method Manual_XLR70 kit for 2× 300-bp paired-end sequencing.

### Bioinformatics analysis

Analysis of the 16S rRNA sequencing data was performed in QIIME^51^. Sequence data from two subjects were excluded from the analysis because of sample contamination. Adaptors, low-quality reads shorter than 200 bp or those with more than 20% low-quality (score of 20) bases, and low-complexity reads (more than 10 of the same consecutive bases) were filtered. Chimeras were detected and removed using UCHIME^52^. High-quality sequences were clustered into OTUs at 97% sequence similarity using USEARCH (version 7.1 http://drive5.com/uparse/). Taxonomic assignment of OTUs was performed based on comparison with a database of curated sequences derived from the Silva Database Project (Release 115, http://www.arb-silva.de)^53^ using RDP Classifiers. The relative abundances of different phyla, genera and OTUs in each sample were calculated and compared before and after the 4-week RS or CS treatments in normal-weight subjects using the Wilcoxon signed-rank test via the SPSS 17.0 software (SPSS Inc., Chicago, IL, USA). A non-metric multidimensional scaling (NMDS) analysis based on the microbiota beta diversity (weighted UniFrac) was performed and visualized in R.

### Statistical analysis

All analyses were performed using the SPSS 17.0 software. Sample size calculations were based on equivalence between treatments, which was defined as a difference in the post-treatment visceral fat area of 6 cm^2^ or more. In our earlier study, the average visceral fat area was 27 ± 5 cm^2^ in healthy, young, normal-weight adults. We estimated that for a two-sided significance level of 0.05 and 80% power, a minimal sample size of 16 was required. To allow for a 20% drop out rate, at least 22 subjects had to be enrolled^54^. The general estimating equation (GEE) was used to model the effects of starch treatment and the effects of time, which accounted for the correlation of repeated measurements within subjects. The final results were presented as the mean ± SEM. Data with an abnormal distribution were expressed as medians with interquartile ranges. A two-sided *p* value less than 0.05 was considered significant.

## Supplementary information

Supplementary information
