# Supplementary material for: Metabolic phenotypes and the gut microbiota in response to dietary resistant starch type 2 in normal-weight subjects: a randomized crossover trial
Source: Sci Rep. 2019 Mar 20;9:4736. doi: 10.1038/s41598-018-38216-9 (PMC6426958; doi:10.1038/s41598-018-38216-9)
Supplement: Supplementary file 1 — Supplementary information [file 41598_2018_38216_MOESM1_ESM.pdf]

Metabolic phenotypes and the gut microbiota in response to dietary resistant starch type 2 in normal-weight subjects: a randomized crossover trial

Lei Zhang[1, 2, †], Yang Ouyang[3, 4, †], Huating Li[1, *], Li Shen[5], Yueqiong Ni[6, 7], Qichen Fang[1], Guangyu Wu[1, 2], Lingling Qian[1, 2], Yunfeng Xiao[8], Jing Zhang[1, 2], Peiyuan Yin[3], Gianni Panagiotou[6, 7], Guowang Xu[3, *], Jianping Ye[1, 9], and Weiping Jia[1, *]

Supplementary Information

Table S1. Nutrients intake at baseline and after 4-week CS or RS supplementation.

Variable	CS		RS		*p* value
	0 week	4 weeks	0 week	4 weeks	
Total energy (kcal)	1578.09±33.51	1560.02±32.39	1548.09±33.51	1536.51±33.88	NS
Protein (g)	63.36±2.08	64.43±2.08	65.46±2.08	64.35±1.94	NS
Fat (g)	41.14±1.53	42.21±1.31	43.11±1.51	42.68±0.92	NS
Cholesterol (mg)	354.29±22.50	348.86±24.53	358.39±23.50	339.81±13.36	NS
Fiber (g)	9.77±0.48	10.04±0.57	9.84±0.58	54.99±6.62***###	<0.001
Carbohydrates (g)	223.29±5.78	223.59±5.27	223.34±6.08	221.52±5.21	NS

Data are presented as mean ± SEM. Significance was determined by GEE model. *p* value was statistical significance between CS effect and RS effect; *** $p < 0.001$, RS 0 week vs. RS 4 weeks, ### $p < 0.001$, RS 4 weeks vs. CS 4 weeks.

Table S2. Summary of serum metabolites of subjects before and after 4-week CS or RS supplementation.

Variable	CS		RS		*p* value
	0 week	4 weeks	0 week	4 weeks	
Isobutyrate (nM)	0.45(0.42,0.59)	0.46(0.31,0.55)	0.63(0.43,0.88)	0.45(0.38,0.56)	NS
Valerate (nM)	0.46(0.40,0.54)	0.43(0.35,0.48)	0.48(0.43,0.63)	0.43(0.38,0.47)	NS
Isovalerate (nM)	0.44(0.37,0.71)	0.41(0.32,0.44)	0.56(0.4,0.68)	0.37(0.29,0.47)	NS
Hexanoate (nM)	1.44(1.27,1.60)	1.15(1.07,1.37)	1.5(1.26,1.67)	1.35(1.21,1.50)	NS
Carnitine C2:0	0.90(0.68,1.27)	0.85(0.74,1.13)	0.97(0.72,1.11)	0.87(0.80,1.03)	NS
Carnitine C3:0	1.01(0.88,1.18)	1.07(0.82,1.21)	0.95(0.82,1.18)	0.92(0.77,1.09)	NS
Carnitine C4:0	1.04(0.80,1.25)	1.09(0.86,1.40)	0.97(0.80,1.50)	1.03(0.87,1.34)	NS
Carnitine C5:0	0.98(0.81,1.40)	1.08(0.81,1.31)	1.07(0.81,1.26)	1.11(0.84,1.24)	NS
Carnitine C10:0	1.22(0.84,1.36)	1.20(0.84,1.46)	0.97(0.77,1.44)	1.17(0.87,1.44)	NS
Carnitine C10:1	0.99(0.86,1.29)	1.22(0.82,1.44)	0.97(0.78,1.28)	1.11(0.91,1.42)	NS
Carnitine C12:0	1.15(0.90,1.33)	1.12(0.83,1.35)	0.96(0.77,1.45)	1.11(0.99,1.31)	NS
Carnitine C12:1	1.02(0.85,1.19)	0.95(0.87,1.19)	1.08(0.90,1.16)	1.05(0.91,1.24)	NS
Carnitine C14:0	1.15(0.90,1.39)	0.98(0.81,1.30)	1.00(0.86,1.16)	1.09(0.99,1.30)	NS
Carnitine C14:1	1.10(0.86,1.24)	1.05(0.77,1.34)	0.90(0.60,1.34)	1.01(0.83,1.32)	NS
Carnitine C18:2	1.05(0.73,1.4)	0.88(0.77,1.53)	0.93(0.71,1.13)	1.01(0.94,1.18)	NS
PC 1-acyl 32:0	1.04(0.92,1.25)	1.03(0.90,1.10)	1.08(0.91,1.17)	1.17(1.03,1.26)	NS
PC 1-acyl 32:1	0.96(0.86,1.29)	0.89(0.80,1.06)	1.04(0.78,1.22)	1.07(0.87,1.27)	NS
PC 1-acyl 34:0	1.13(0.77,1.34)	0.94(0.80,1.20)	1.22(0.93,1.38)	1.09(1.02,1.25)	NS
PC 1-alk 34:1	1.16(0.91,1.47)	1.17(0.87,1.29)	1.09(0.93,1.31)	1.05(0.97,1.24)	NS
PC 1-alk 34:2	1.09(0.87,1.31)	1.05(0.92,1.18)	1.15(1.04,1.28)	1.22(1.06,1.33)	NS
Indoxyl sulfate	1.22(1.02,1.74)	0.83(0.59,1.38)	1.07(0.86,1.57)	1.29(1.05,1.77)	NS
Indolepropionate	0.22(0.13,0.33)	0.52(0.31,0.75)	0.27(0.17,0.35)	0.22(0.17,0.33)	NS
p-Cresol sulfate	0.91(0.75,1.52)	0.82(0.39,1.79)	0.67(0.35,1.14)	1.06(0.62,1.65)	NS
Phenylacetylglutamine	1.03(0.87,1.33)	1.13(0.44,1.73)	1.02(0.83,1.71)	1.33(0.94,1.65)	NS
Methionine	1.07(0.96,1.35)	1.10(0.96,1.15)	1.08(0.96,1.22)	1.09(1.02,1.19)	NS
5-oxoproline	1.01(0.89,1.10)	1.06(0.98,1.13)	1.00(0.76,1.12)	1.04(0.96,1.15)	NS
Palmitic acid	1.09(0.72,1.55)	1.01(0.88,1.31)	1.27(1.00,1.55)	0.97(0.80,1.29)	NS
Phenylalanine	0.99(0.82,1.13)	0.94(0.86,1.12)	1.07(0.89,1.19)	1.07(0.98,1.15)	NS

Data are presented as median (interquartile range), and the data are log transformed before analysis; Significance was determined by GEE model. *p* value was statistical significance between CS effect and RS effect.

Table S3. Glucose metabolism before and after 4 weeks' RS or CS supplementation in normal-weight subjects.

Variable	CS		RS		*p* value
	0 week	4 weeks	0 week	4 weeks	
Blood glucose 0 min (mmol/l)	4.86±0.09	4.84±0.10	4.78±0.07	4.85±0.10	NS
Blood glucose 30 min (mmol/l)	7.58±0.19	7.69±0.23	7.49±0.21	7.80±0.22	NS
Blood glucose 60 min (mmol/l)	6.88±0.33	6.57±0.28	7.04±0.34	6.79±0.32	NS
Blood glucose 120 min (mmol/l)	5.84±0.25	5.54±0.16	5.64±0.21	6.03±0.28	NS
Insulin 0min (μU/ml)	8.33±0.95	8.13±1.03	8.43±1.10	8.31±0.76	NS
Insulin 30 min (μU/ml)	56.11±6.17	56.98±7.57	55.29±6.82	69.7±6.90*$^{\#}$	0.050
Insulin 60 min (μU/ml)	49.21±4.46	48.27±6.87	47.21±4.57	59.50±6.86	NS
Insulin 120 min (μU/ml)	31.70±2.49	28.98±2.62	30.16±2.57	38.48±4.54	NS
C peptide 0min (ng/ml)	1.67±0.11	1.79±0.15	1.69±0.12	1.78±0.09	NS
C peptide 30min (ng/ml)	5.56±0.44	5.72±0.48	5.50±0.47	6.59±0.45***$^{\#\#}$	0.008
C peptide 60min (ng/ml)	6.59±0.42	6.56±0.50	6.50±0.42	7.01±0.38	NS
C peptide 120min (ng/ml)	5.74±0.40	5.56±0.49	5.64±0.41	6.44±0.49	NS
GIR (mg*kg-1*min-1)	7.97±0.58	8.42±0.72	8.27±0.51	8.09±0.51	NS
HOMA-IR	1.75±0.20	1.69±0.20	1.81±0.24	1.81±0.18	NS
HOMAβ (%)	119.18±8.32	117.69±9.34	132.67±17.71	127.00±11.40	NS

Data are presented as mean ± SEM. Significance was determined by GEE model. p value was statistical significance between CS effect and RS effect; * $p < 0.05$, *** $p < 0.001$, RS 0 week vs. RS 4 weeks, $^{\#}$ $p < 0.05$, $^{\#\#}$ $p < 0.01$, RS 4 weeks vs. CS 4 weeks..

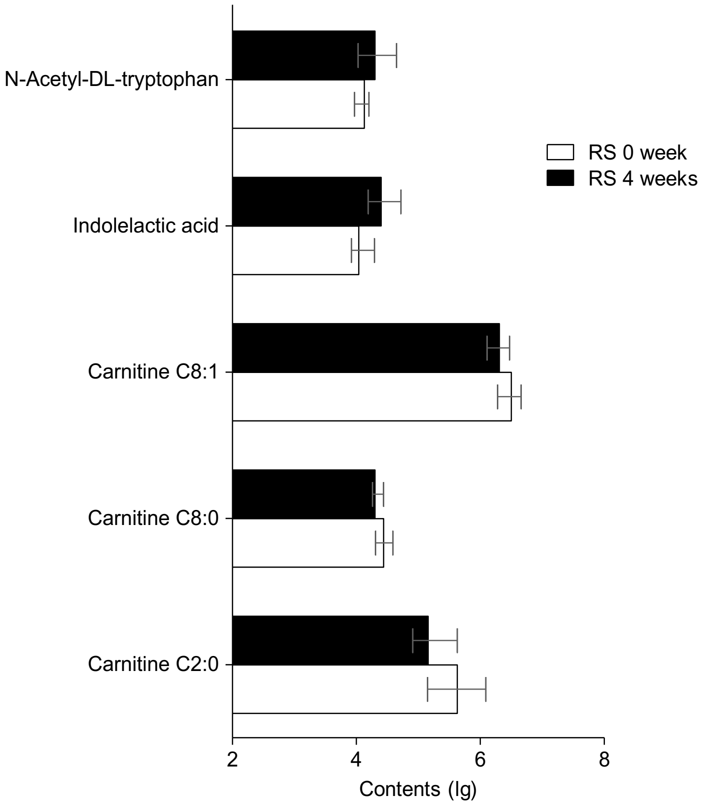

Fig. S1. Urine metabolomics before and after 4-week RS supplementation in normal-weight subjects. Urine metabolomics before and after 4-week RS supplementation were analyzed. Only the urine metabolomics whose content were significantly changed before and after 4-week RS supplementation in individuals with normal weight were displayed (n=19). Data represent median (interquartile range), and the data are log transformed before analysis; Significance was determined by GEE model. All were $p < 0.05$.

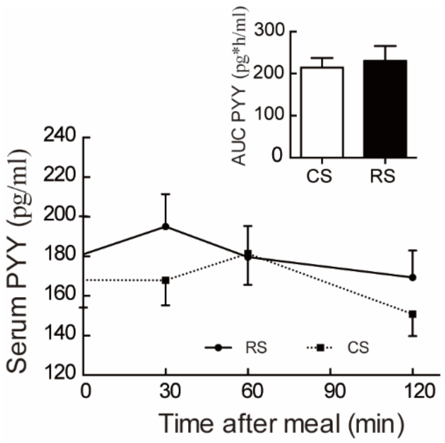

Fig. S2. Serum PYY levels and AUC after a standardized meal in participates after 4-week RS or CS supplementation. Data are presented as mean ± SEM.

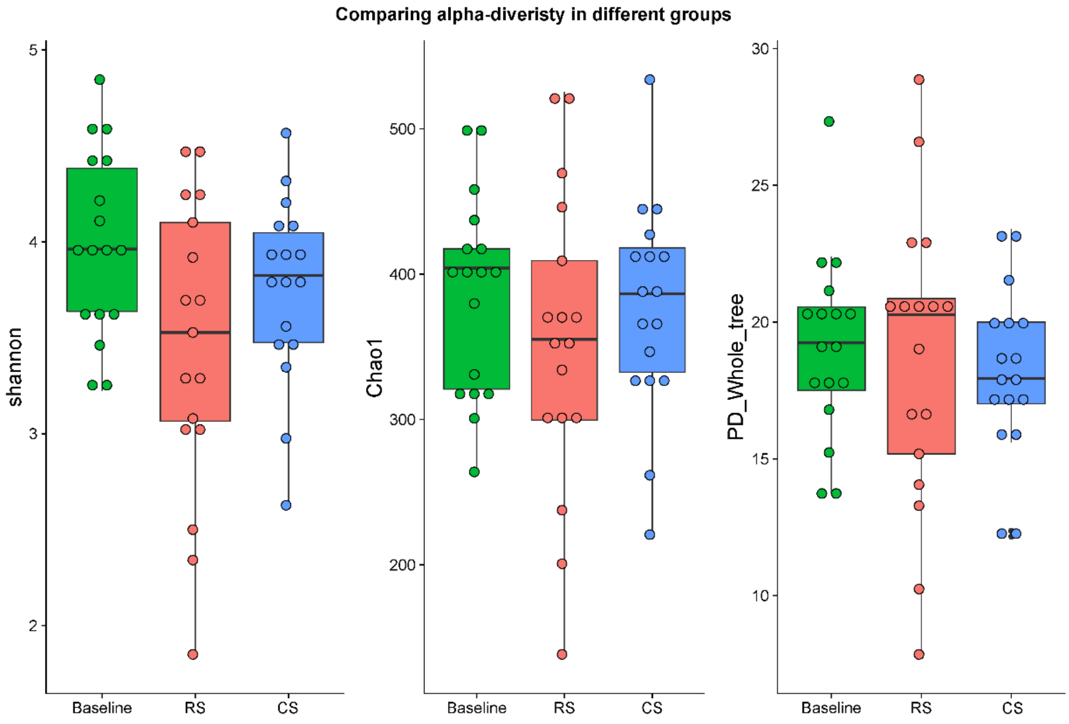

Fig. S3. Alpha diversity at baseline, after 4-week RS supplementation and after 4-week CS supplementation were analyzed (n=17). Data represent median (interquartile range); Significance was determined by Mann-Whitney test between groups.

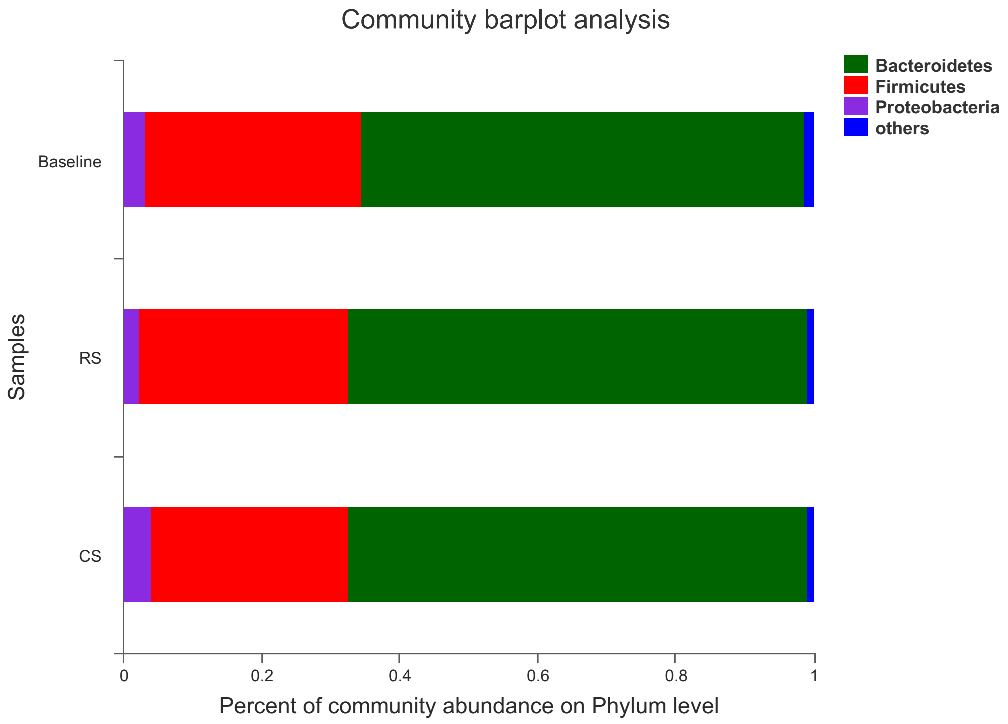

Fig. S4. Percent of community abundance on phylum level at baseline, after 4-week RS supplementation and after 4-week CS supplementation.

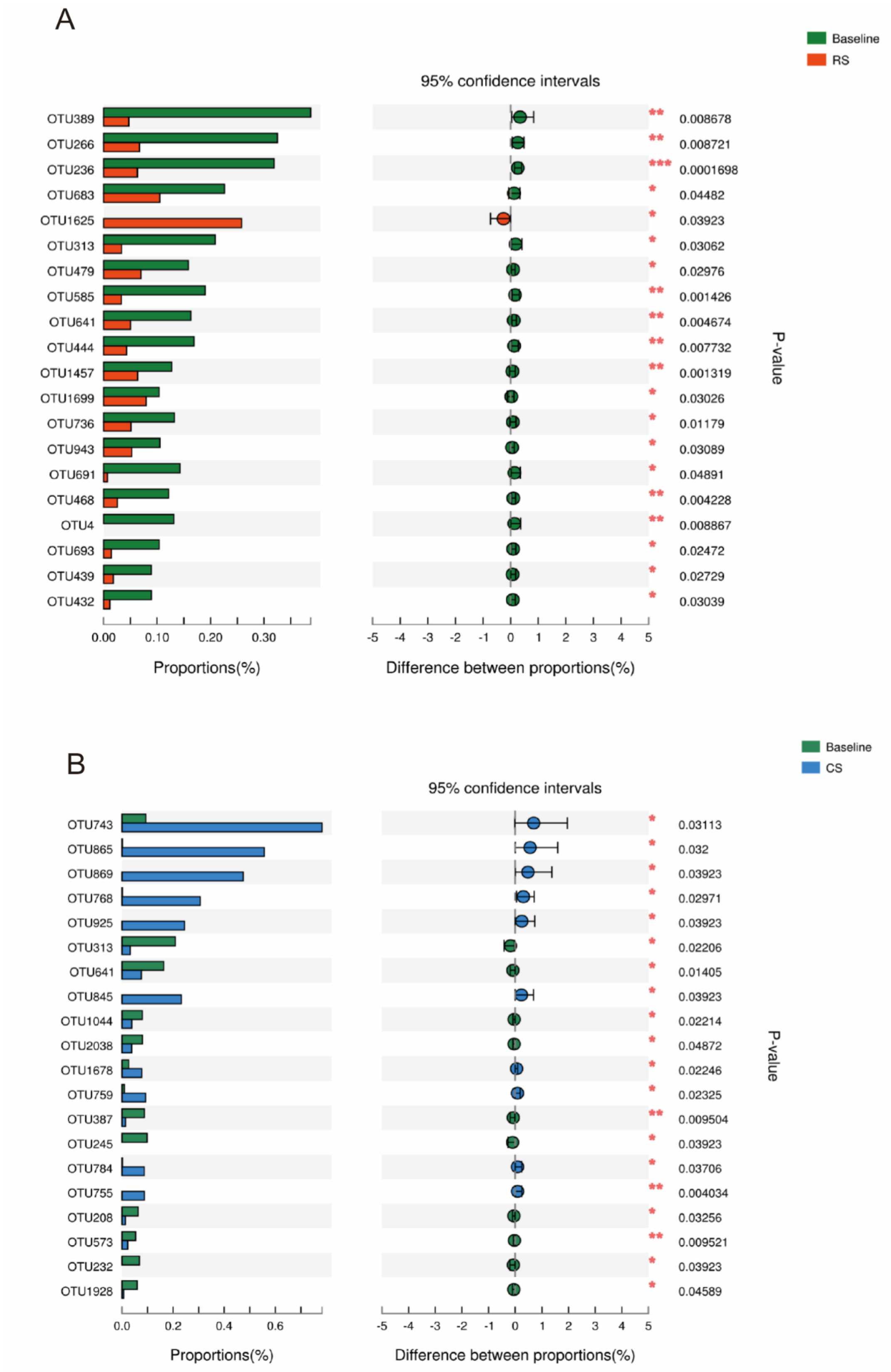

Fig. S5. The change of gut microbial composition after RS supplementation at the OTU level. The differences of bacterial abundances at the OTU-level before and after 4-week RS (A) or CS (B) intake in normal-weight individuals were analyzed, only the top 20 OTU whose abundances were significantly changed (* $p < 0.05$, ** $p < 0.01$, *** $p < 0.001$, Wilcoxon signed-rank test) were displayed (n=17). Mean abundances of all samples at baseline or after supplementation were used.

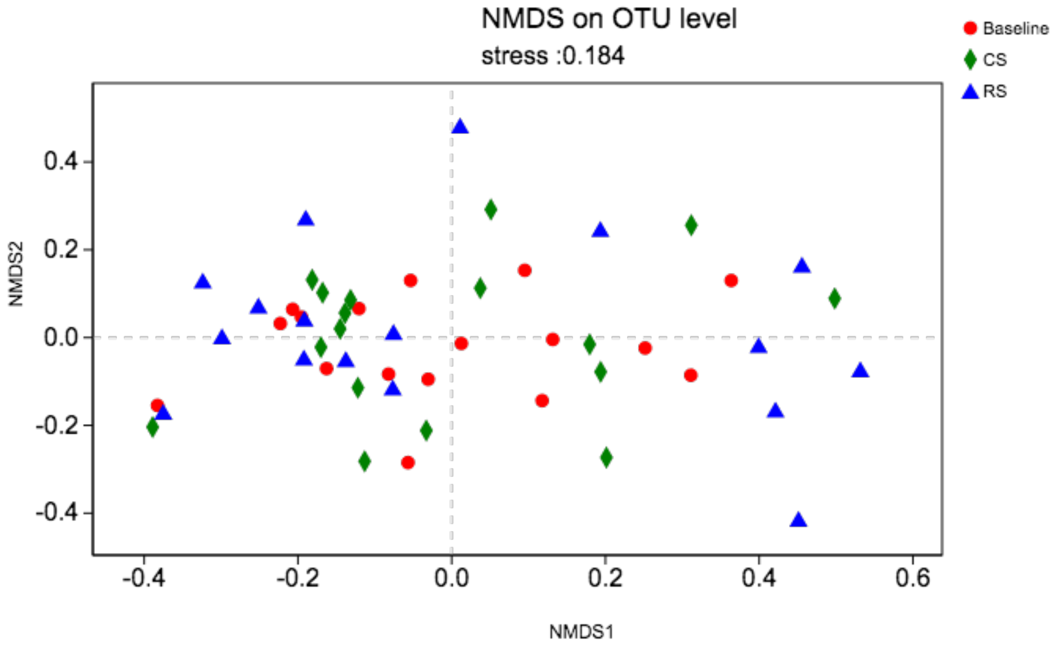

Fig. S6. Non-metric multidimensional scaling (NMDS) ordination plot based on OTU-level microbial beta diversity (UniFrac distances).
